# A Pilot Trial of Two-Fraction Stereotactic Body Radiation Therapy for Localized Prostate Cancer

**DOI:** 10.7759/cureus.100519

**Published:** 2025-12-31

**Authors:** Xinglei Shen, Matthew Chen, Ying Cao, Amanda Schroeder, Leah Miller, Jay C Shiao

**Affiliations:** 1 Radiation Oncology, University of Kansas Medical Center, Kansas City, USA

**Keywords:** clinical trial, pilot, prostate cancer, stereotactic body radiation therapy (sbrt), ultra-hypofractionated radiation

## Abstract

Objectives: Ultra-hypofractionated radiation is a widely adopted treatment for low- and intermediate-risk prostate cancer and is typically given for a duration of five treatments in the United States. There is interest in developing a shorter two-treatment course. We conducted a pilot study of two-fraction ultra-hypofractionated radiation therapy for low- to intermediate-risk prostate cancer to determine safety and preliminary efficacy.

Methods: A total of 12 patients were prospectively enrolled in a single-arm pilot study of two-fraction ultra-hypofractionated radiation therapy. Patients were treated with 12.5 Gy in two fractions separated by 2-3 days. All patients had placement of a rectal spacer. Biochemical response was evaluated with prostate-specific antigen (PSA) monitoring, with recurrence defined using the American Society for Therapeutic Radiology and Oncology (ASTRO) Phoenix criteria. Toxicity was graded using the Common Terminology Criteria for Adverse Events (CTCAE) v5.0.

Results: Median follow-up was 4.8 years. This cohort consisted of National Comprehensive Cancer Network (NCCN) intermediate-risk patients (seven NCCN favorable intermediate and five NCCN unfavorable intermediate). The median prostate volume was 30 cc. The median PSA nadir was 0.14 (range <0.01 to 0.82). No patient experienced a biochemical recurrence. No patient experienced grade 2 or worse gastrointestinal (GI) acute or late toxicity. Three of the 12 patients experienced acute grade 2 genitourinary (GU) toxicity, and two of the 12 patients experienced late grade 2 toxicity. No patient experienced grade 3 toxicity.

Conclusions: Two-fraction ultra-hypofractionated radiation to 25 Gy is tolerable with a low rate of long-term toxicity and promising biochemical control. Further research is indicated to evaluate two-fraction regimens for prostate radiation.

## Introduction

Radiation therapy is well-established as a common primary curative treatment for localized prostate cancer [1]. With improvements in technology and completion of multiple randomized trials, external beam radiation treatment courses have shortened dramatically, from conventional fractionation (usually 39-44 treatments) to moderately hypofractionated regimens (20-28 treatments) to stereotactic body radiation therapy (SBRT) (five treatments or less). The recently published PACE-B (Prostate Advances in Comparative Evidence B) trial provided support for a five-fraction SBRT course as a standard radiation option in patients with low- to intermediate-risk prostate cancer [2]. Reducing treatment duration, if curative efficacy and the safety profile of radiation therapy can be maintained, is more convenient for patients, reduces the impact of cancer therapy on work and other aspects of life, and also reduces overall costs for the patient and the healthcare system.

High-dose rate (HDR) brachytherapy is another form of primary treatment for localized prostate cancer, and a two-fraction regimen is commonly used with excellent long-term patient outcomes [3]. There is interest in delivering a similar regimen using SBRT. This would provide patients with faster but similarly effective treatment while avoiding multiple brachytherapy procedures and anesthesia. However, there is little published data on the safety and efficacy of two-fraction SBRT. This pilot trial was designed to help fill this current knowledge gap.

## Materials and methods

This was a single-arm prospective trial. Patients with National Comprehensive Cancer Network (NCCN) low- or intermediate-risk prostate cancer (clinical stage T1c-T2bN0M0, Gleason score 6-7, PSA <20 ng/mL) and prostate size <60 cc were eligible [1,4]. The primary objective of this pilot trial was to describe the rates of toxicity of this two-fraction SBRT regimen. Secondary objective included biochemical control based on the American Society for Therapeutic Radiology and Oncology (ASTRO) Phoenix criteria [5]. This trial was approved by the University of Kansas Medical Center Institutional Review Board (IRB) (141963) and registered on Clinicaltrials.gov (NCT03486821). Trial enrollment occurred from 3/22/2018 through 7/30/2021. Informed consent was obtained from each participant prior to enrollment, and the study was conducted in accordance with good clinical practice (GCP).

Patients received 12.5 Gy × 2 fractions completed within a total of four days, using a conventional linear accelerator. This dose was chosen because it provides an equivalent dose in 2 Gy fractions (EQD2) to the tumor that is higher than other commonly-used regimens (assuming a/b=1.5, EQD2 is 90.6 Gy for 36.25 Gy/5 fractions, 100 Gy for 25 Gy/2 fractions), and comparable EQD2 for normal tissue (a/b=3, EQD2 is 74.3 Gy for 36.25/5 and 77.5 Gy for 25/2). Fiducial markers and a per-rectal hydrogel spacer were placed for all patients. Clinical target volume (CTV) was defined as the entire prostate; inclusion of up to 1 cm of seminal vesicles was optional. Planning target volume (PTV) included a 5 mm circumferential expansion of the CTV, and 3-5 mm posteriorly. Protocol dose constraints are summarized in Table 1. Cone-beam computed tomography (CT) was used for daily image guidance. Androgen deprivation therapy (ADT) was allowed at the physician's discretion.

**Table 1:** Protocol dose constraints PTV: Planning target volume; Vx: Volume receiving at least x% of the prescribed dose; Dx: Dose delivered to x% of the volume

Structure	Constraint
PTV	V100 ≥ 95%, Minimal dose to 0.05 cc ≥ 95%, Global maximum ≤ 107%
Rectum	V100 < 3 cc, V80 < 8 cc, D100 < 50%
Bladder	D10 < 90%, D50 < 50%

Descriptive statistics were used to summarize patient outcomes. Microsoft Excel (Microsoft Corp., Redmond, USA) was used for statistical analyses [6].

## Results

A total of 12 patients were enrolled and completed protocol radiation therapy; four also received short-term ADT (Table 2). All patients had NCCN intermediate-risk disease (seven NCCN favorable intermediate and five NCCN unfavorable intermediate); the median prostate size was 30 cc (range 18-50), and the median baseline International Prostate Symptom Score (IPSS) score was 6 (range 1-22) [7].

**Table 2:** Baseline characteristics of enrolled participants NCCN: National Comprehensive Cancer Network; IPSS: International Prostate Symptom Score

Patient Characteristic	N (%)
Age (years)	
Median (range)	67 (59-81)
Race	
White	11 (91.7)
Black or African American	1 (8.3)
Ethnicity	
Not Hispanic or Latino	12 (100)
Clinical T stage	
T1c	11 (91.7)
T2a	1 (8.3)
Baseline PSA (ng/mL)	
Median (range)	7.38 (5.36-14.87)
Gleason score	
6	1 (8.3)
3+4	8 (66.7)
4+3	3 (27.3)
Risk group (NCCN)	
Favorable intermediate-risk	7 (58.3)
Unfavorable intermediate-risk	5 (41.7)
Prostate volume (cc)	
Median (range)	30 (18-50)
IPSS score at baseline	
Median (range)	6 (1-22)
Androgen deprivation therapy	
Yes	4 (33.3)
No	8 (66.7)

**Figure 1:**
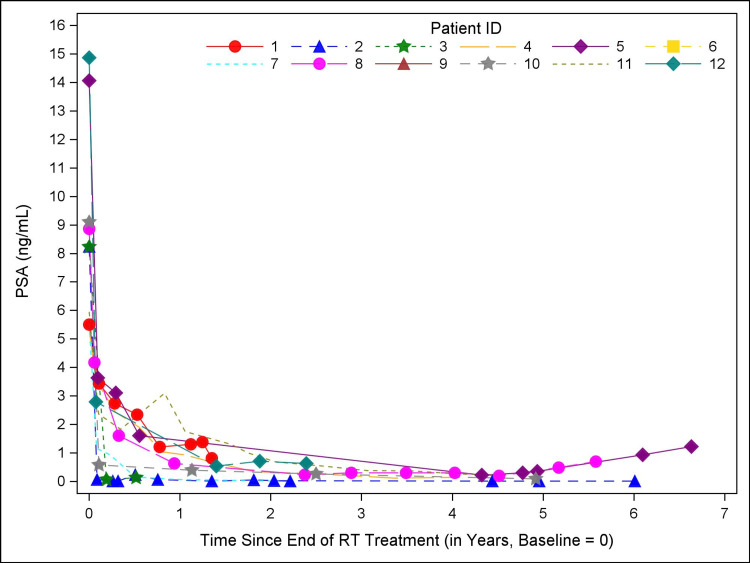
PSA kinetics Trajectory of PSA (ng/mL) after treatment with two-fraction SBRT. Baseline PSA (pre-treatment) is defined as time 0. Each individual patient is represented by a unique symbol. No patient met criteria for biochemical recurrence. The median PSA nadir is 0.14 (range <0.01 to 0.82). PSA: Prostate-specific antigen; RT: Radiation therapy; SBRT: Stereotactic body radiation therapy

Median follow-up was 4.8 years (range 0.6-6.6). All patients had at least two years of follow-up after completing treatment, except one patient who died at seven months from an unknown cause. No patient experienced biochemical recurrence. PSA trajectory is summarized in Figure 1. Median PSA nadir was 0.14 (range undetectable to 0.82).

No patient experienced grade 2 or higher gastrointestinal (GI) toxicity. Three patients experienced Common Terminology Criteria for Adverse Event (CTCAE) v5.0 grade 2 urinary toxicity within three months of finishing SBRT (one with urinary tract pain that resolved with steroids, two with urinary tract obstruction that resolved with tamsulosin) [8]. Two different patients experienced grade 2 late urinary toxicity (one patient with hematuria who declined further work-up and died from an unrelated cause, one with urinary tract obstruction and frequency managed with tamsulosin). There was no grade 3 or higher urinary toxicity.

## Discussion

In this pilot prospective trial, 12 patients with intermediate-risk prostate cancer were treated using a conventional linear accelerator, delivering 25 Gy in two fractions over 3-4 total days. After a median follow-up of almost five years, the median nadir PSA achieved was 0.14, and no patient developed a biochemical recurrence. Three patients experienced acute grade 2 urinary toxicity that completely resolved, and two patients developed late grade 2 urinary toxicity. Although not mandated by protocol, all patients had hydrogel spacer placement before treatment. No patient developed grade 2 or higher acute or late GI toxicity. It is possible that we could have seen a higher rate of rectal complications had we not used a rectal spacer, and this should be considered in future trial designs.

While five-fraction SBRT is commonly used for localized prostate cancer, there is limited data on two-fraction SBRT regimens. Udovicich et al. published a report on 56 intermediate-risk patients treated in two separate phase 2 trials in Toronto, using two weekly fractions (26 Gy to the CTV and 22 Gy to the PTV) and an endorectal immobilization device during treatment [9]. Similar to our findings, Udovicich et al. found a median nadir PSA of 0.11 ng/mL and a low rate of biochemical failure (the eight-year rate was 6.4%). Low rates of GI and urinary toxicity were also reported.

The SABR-Dual (Stereotactic Ablative Radiotherapy-Dual) trial reported acute toxicity results from 20 patients with a median follow-up of eight months [10]. This trial utilized a dose of 27 Gy in two fractions (administered at least 72 hours apart) to the PTV-prostate. All patients had a hydrogel spacer placed and took 2 mg of dexamethasone before each radiation treatment. No patient developed grade 2 or higher GI toxicity; however, 10% experienced grade 2 urinary toxicity with no grade 3 or higher toxicity.

The current trial is limited by its small sample size. Further data are necessary to confirm these findings in a larger trial. However, our trial adds to the limited existing literature, suggesting that a two-fraction SBRT regimen delivered via a conventional linac is safe and demonstrates promising efficacy for intermediate-risk prostate cancer. The dose in our trial is the lowest among the reports but was chosen rationally to deliver a biological dose that is higher than the commonly used five-fraction SBRT regimen of 36.25 Gy while keeping a similar biological dose to organs at risk. We also demonstrate that delivering a two-dose regimen using a routine SBRT schedule (e.g., every other day), without an endorectal immobilization device (as in the Toronto trial) or steroid medication (like in the SABR-Dual trial), appears to be well-tolerated.

With these promising data, several randomized trials have begun to compare two-fraction versus five-fraction SBRT. Two of these trials - HERMES (Hypofractionated Expedited Radiotherapy for Men With Localized Prostate Cancer) [11] and FORT (Randomized Trial of Five or Two MRI-Guided Adaptive Radiotherapy Treatments for Prostate Cancer) [12] - utilize magnetic resonance (MR)-guided SBRT, while SABR-Dual uses a conventional linac [10]. FORT employs the same dose as ours (25 Gy in two fractions). The completion of these trials will determine whether two-fraction SBRT can potentially provide similarly effective and safe treatment for localized prostate cancer as HDR brachytherapy, while avoiding the more invasive HDR procedures and anesthesia.

## Conclusions

Two-fraction linear accelerator-based ultra-hypofractionated radiation therapy at 12.5 Gy × 2 is very well tolerated and provides excellent cancer control. These data support the further development of two-fraction regimens for the treatment of intact prostate cancer.
